# Development of a rapid MALDI-TOF MS based epidemiological screening method using MRSA as a model organism

**DOI:** 10.1007/s10096-017-3101-x

**Published:** 2017-09-18

**Authors:** Åsa Lindgren, Nahid Karami, Roger Karlsson, Christina Åhrén, Martin Welker, Edward R. B. Moore, Liselott Svensson Stadler

**Affiliations:** 1 Department of Infectious Diseases, Institute of Biomedicine, Sahlgrenska Academy at the University of Gothenburg, Gothenburg, Sweden; 2 Centre for Antibiotic Resistance Research (CARe) at University of Gothenburg, Gothenburg, Sweden; 3 Nanoxis Consulting AB, Gothenburg, Sweden; 4 Swedish Strategic Programme Against Antibiotic Resistance, Region Västra Götaland, Gothenburg, Sweden; 5 Unit Microbiology, R&D Microbiology, bioMérieux SA, La Balme Les Grottes, France; 6 Culture Collection University of Gothenburg (CCUG), Gothenburg, Sweden

## Abstract

In this study we present a method using whole cell MALDI-TOF MS and VITEK MS RUO/SARAMIS as a rapid epidemiological screening tool. MRSA was used as a model organism for setting up the screening strategy. A collection of well-characterised MRSA strains representing the 19 most common Pulsed-Field Gel Electrophoresis (PFGE)-types in the region of South-West Sweden for the past 20 years was analysed with MALDI-TOF MS. A total of 111 MRSA strains were used for creating 19 PFGE-specific Superspectra using VITEK MS RUO/SARAMIS. Prior to performing the final analysis, the 19 Superspectra were combined into ten groups displaying similar peak patterns, hereafter named “MALDI-types”. Two-hundred fifty-five MRSA strains were analysed to test the constructed Superspectra/MALDI-type database. Matches to the Superspectra above a threshold of 65% (corresponding to the number of matched peaks in the Superspectrum) were considered as positive assignment of a strain to a MALDI-type. The median peak matching value for correct assignment of a strain to a MALDI-type was 78% (range 65.3–100%). In total, 172 strains (67.4%) were assigned to the correct MALDI-type and only 5.5% of the strains were incorrectly assigned to another MALDI-type than the expected based on the PFGE-type of the strain. We envision this methodology as a cost-efficient step to be used as a first screening strategy in the typing scheme of MRSA isolates, to exclude epidemiological relatedness of isolates or to identify the need for further typing.

**Electronic supplementary material:**

The online version of this article (10.1007/s10096-017-3101-x) contains supplementary material, which is available to authorized users.

## Introduction

Whole cell Matrix-Assisted Laser Desorption/Ionisation - Time Of Flight Mass Spectrometry (MALDI-TOF MS) has been a major breakthrough for microbial identification in the clinical microbiology laboratory [1, 2]. It has been shown to be able to process microbiological identification much faster and in some cases with higher resolution than traditional phenotyping. The combination of a rapid analysis and low cost makes it a valuable tool for processing large numbers of samples.

VITEK MS Plus (bioMérieux, Marcy l’Etoile, France) is one of the commercial systems used for species identification with MALDI-TOF MS in microbiological diagnostics. It consists of two databases that use different software, the closed IVD database, mainly used for routine identification, and the Research Use Only (RUO)/SARAMIS databases. The RUO/SARAMIS system is an open database in which the user can add spectra and curate the database(s). It employs the concept of Superspectra for identification, which is a composite spectrum containing conserved and specific peaks identified in a population spectra from well-characterised reference strains [3]. Peaks are weighted to account for specificities at different taxonomic levels, for example, family, genus, and species levels or like in this study, the type level.

The successful implementation of MALDI-TOF MS in the clinical microbiology laboratory and the ease and speed of the analysis have spurred further investigations of how to exploit MALDI-TOF MS for more detailed analyses, i.e. at sub-species levels. There are increasing numbers of studies investigating the potential levels of resolution able to be elucidated by MALDI-TOF MS for epidemiological purposes as well as for typing with regard to virulence or antibiotic resistance, such as the typing of *Clostridium difficile* [4], *Haemophilus influenza* type B capsule typing [5], outbreak typing of Shiga-toxigenic *Escherichia coli* [6] and subtyping of *Streptococcus equi* [7].

Methicillin resistant *Staphylococcus aureus* (MRSA) infections are a worldwide health problem and a frequent cause of both hospital- and community-associated infections [8]. Fast and reliable epidemiological typing of MRSA infections is crucial for identifying chains of transmission and to identify and control outbreaks. Molecular methods such as Pulsed-Field Gel Electrophoresis (PFGE), *Staphylococcus* protein A (*spa*) typing and Multi-Locus Sequence Typing (MLST) have been widely used to type MRSA isolates [9, 10]. PFGE has excellent discriminatory power and has a long history of being the gold standard in outbreak investigations. More recently, Next Generation Sequencing (NGS) based techniques are increasingly being used [11, 12]. There is a good degree of concordance between these methods, although the levels of resolution differ as well as the amount of hands-on-time needed [13]. Despite the rapid development in NGS technology, interpretation of data still requires substantial expertise. For everyday epidemiological surveillance a combination of methods used in parallel or sequentially may be most time and resource efficient.

A number of studies have made attempts to use MALDI-TOF MS for epidemiological typing of MRSA [14–16]. The main focus of these studies has been to find specific biomarkers representing subsets of MRSA strains. In addition there have been studies attempting to find markers specific for MRSA, to be able to use MALDI-TOF MS for distinguishing between MRSA and Methicillin sensitive *S. aureus* (MSSA) strains [17].

In this study we present a method using whole cell MALDI-TOF MS without prior protein extraction, creating Superspectra with VITEK MS RUO/SARAMIS for rapid epidemiological typing of MRSA.

## Material and methods

### Selection of strains

The first MRSA strains isolated from each patient in South-West Sweden have been stored in the Culture Collection University of Gothenburg (CCUG; www.ccug.se) since 1983 together with patient epidemiological data. The strains have been identified as MRSA, according to routine laboratory protocols, and have, until 2016, been typed with PFGE. In addition, representative strains of each PFGE-type have been *spa-*typed until this method was implemented as routine in 2003 (Supplementary information, Table S1).

Altogether, 111 strains representing the 19 most common MRSA PFGE-types were selected for construction of a MALDI-TOF MS database consisting of Superspectra specific for individual PFGE-types. These so-called “*Superspectra strains*” represented one to six subtypes within each PFGE-type, 60 *spa*-types, 10 *spa*-clonal complexes (CC) and 11 MLST-CC (Table 1). The PFGE-type was determined by cluster analysis using the BioNumerics software version 7.1 (Applied Maths NV, Sint-Martens-Latem, Belgium) with the Dice coefficient for calculating pair-wise similarities and the UPGMA algorithm for constructing dendrograms of estimated relatedness. For creation of the Superspectra, four to ten strains of a particular PFGE-type, but of different PFGE-subtypes, were selected (*Superspectra strains*, Table 1). For the selected strains the maximal variation within each PFGE-type was set at 70% similarity and inclusion of strains with identical PFGE-profile were avoided.

**Table 1:** Characteristics of the 111 *Superspectra strains* used for construction of the Superspectra database, representing the 19 most common PFGE-types in the region

MALDI-type	PFGE-type	PFGE-subtypes (n)	*spa-CC (n)*	MLST-CC	Strains (n)
M1	B	2	CC-012(5), n.d.^a^(1)	30	6
M2	BK	6	CC-790/005(6)	22	6
	I	2	CC-790/005(5)	22	5
M3	C	2	CC-008(1)	8	6
			CC-324(5)	72	
M4	Cy	1	CC-012(3)	30	4
			n.d.^a^(1)	n.d.	
	SM	1	CC-012(6)	30^d^	6
M5	D	1	CC-441/437(5)	59	5
	J2	1	CC-441/437(3)	59	5
			t5773^b^(1), t6750^b^(1)	n.d.	
	R	5	t127^c^(2)	1	6
			CC-008(3)	6/8	
			n.d.^a^(1)	n.d.	
M6	F	1	CC-015(6), t026^b^(1), t390^b^(1)	45^d^	8
M7	J3	3	CC-002(3), t7284^b^(1)	5	4
	J	1	CC-345/359(5)	1	6
			n.d.(1)	n.d.	
	L	2	CC-002(5)	5	5
	S	2	CC-002(3)	5	5
			CC-008(1)	6/8	
			t535^b^(1)	n.d.	
	A	3	CC-002(5)	5	5
M8	K	1	CC-008(5), t400^c^(1)	8^d^	6
	M	1	CC-008(6)	8	6
M9	P	1	CC-345/359(6), t416^b^(1)	80^d^	10
			t10892^c^(1), t004^c^(1), n.d.^a^(1)	n.d.	
M10	T	6	CC-448/690(6)	88	7
			n.d.^a^(1)	n.d.	

^a^ *spa-*type not determined

^b^Excluded from cluster analysis due to less than four *spa*-repeats

^c^Singletons

^d^The indicated MLST-CC was determined by MLST analysis. The reminders were deducted from the *spa*-type (Ridom *spa* server)

For subsequent testing of the performance of the created database, two sets of strains were tested. These so-called “*test strains*” (Table 2), included 146 strains isolated from routine clinical and MRSA screened samples from patients in the Gothenburg region between October 2014 and April 2015. Secondly, 109 MRSA strains of different PFGE-types and subtypes were arbitrarily selected from the CCUG MRSA strain collection in order to test a more diverse collection of strains. MLST data were available for 59 of these 255 strains, for the remainder the most common MLST-CC were deduced from the *spa*-type, using the Ridom Spaserver (www.spa.ridom.de). The 255 MRSA *test strains* were of 25 different PFGE-types with 1-17 subtypes/PFGE-type, 102 s*pa*-types, 12 *spa*-CC and 18 MLST-CC (Table 2).

**Table 2:** Characteristics of 255 *test strains*, expected assignment to MALDI-type according to PFGE-type

Expected MALDI-type	PFGE-type	PFGE-subtypes (n)	*spa-CC (n)* ^*a*^	MLST-CC^b^	Strains (n)
M1	B	7	**CC-012(14)**	30	15
M2	BK/I	12/9	**CC-790/005(30)**, t309^c^(1)	22	31
			t272^c^(1)	121	1
			t1233^c^(1), t3507^c^(1), n.d.^d^(3)	n.d.	5
M3	C	1	**t3196** ^**e**^ **(1)**	72	1
M4	Cy/SM	2/13	**CC-012(18),** t748^e^(1)	30	19
			CC-091(1)	15	1
M5	D/J2/R	1/2/17	t127^c^(15)	1	15
			CC-002(1)	5	1
			**CC-008(14)**	6/8	14
			CC-091(1)	7	1
			CC-441/437(3)	59	3
			t504^e^(1), t1178^e^(1), t1784^e^(3), n.d.^d^(2)	n.d.	7
M6	F	13	**CC-015(12),** t2363^c^(1), t390^e^	45	15
			(1),t026^e^(1) t370^c^(1)	n.d.	1
M7	J3/L/S/J/A	6/4/0/6/6	**CC-345/359(5),** t7358^c^(1)	1	6
			CC-002(20), CC-010(1), CC-071(2), t777^e^(1)	5	24
			CC-008(1)	6	1
			t2315^c^(1), t5981^c^(1), t7284^c^(1), t8503^c^(1), n.d.^d^(5)	n.d.	9
M8	K/M	9/5	**CC-008(28)**	8	28
			CC-202(1)	(ST93)	1
			n.d.^d^(1)	n.d.	1
M9	P	10	**CC-345/359(14)**	80	14
M10	T	8	**CC-448/690(20)**	88	20
			CC-790/005(1)	22	1
			CC-202(1), t11550^c^(1), n.d.^d^(1)	n.d.	3
	Other^f^	12	t386^e^(1)	1	1
			CC-008(2)	8	2
			CC-091(2)	15	2
			CC-012(1)	30	1
			CC-345/359(3)	97	3
			t314^c^(1)	121	1
			t034^c^(2)	398	2
			t375^e^(1)	509	1
			t991^e^(2), t15556^c^(1), n.d.^d^(1)	n.d.	4

^a^The dominating *spa-*CC (founder *spa*-type indicated in the table) within each group is marked with bold letters

^b^The indicated MLST-CC was determined by MLST analysis. The reminders were deducted from the *spa*-type (Ridom *spa* server)

^c^Singletons

^d^ *spa*-CC not determined

^e^Excluded from cluster analysis due to less than four repeats

^f^Others refer to strains with PFGE-types for which no Superspectrum were present in the database

### MALDI-TOF MS

MALDI-TOF MS analysis was performed with intact cell biomass obtained from overnight cultures at 37 °C on horse blood agar plates. Samples were spotted in four replicates, on disposable target plates (bioMérieux) and overlaid with 1 μl VITEK MS-CHCA matrix (α-cyano-4-hydroxycinnamic acid, bioMérieux). Beforehand, strains had been tested with incubation in 30 °C or 37 °C as well as on target addition of 0.5 μl VITEK MS-FA (Formic acid, bioMérieux) and subsequently 1 μl VITEK MS-CHCA. For repeated testing of four strains the mean (median) number of peaks were 156.5 (156.6) and 160.3 (164) using 30 °C or 37 °C, respectively; hence, since 37 °C is the standard incubation temperature used in the clinical laboratory, this temperature was chosen. FA did not increase the resolution of the spectra, and was thus not included in the final protocol. Mean (median) number of peaks for the four tested strains for FA-treated and untreated samples was 160.1 (160) and 160.3 (164), respectively.

MALDI-TOF MS analysis was performed using a VITEK MS RUO instrument (bioMérieux), in the range of 2000-20,000 m/z, with a laser frequency of 50 Hz, an acceleration voltage of 22 kV and an extraction delay time of 83 ns. Spectra were acquired in automatic mode by accumulating 100 profiles of five laser shot cycles each using the auto quality control of Launchpad 2.9. Spectral data were automatically processed and exported as peak lists for analysis in SARAMIS. An exclusion list with horse blood agar specific peaks was applied to the spectra to avoid non-bacterial peaks within the spectra. The *E. coli* strain CCUG 10979 was used as a control strain in each run.

Three of the four replicate spectra of each strain were selected for further analysis in each run. First, all spectra with less than 100 peaks were excluded, the remaining replicate spectra of each strain were then compared using the Pearson correlation in the SARAMIS software. Spectra with less than 85% similarity to at least two of the other replicates of the same strain were considered to be outliers and also removed from further analysis. If all four replicate spectra showed ≥85% similarity, as they did in 75% of the cases, the three most similar spectra were selected for further analyses. If three good quality replicate spectra could not be obtained from one run the strain was retested and analysed according to the above procedure; hence, three replicate with ≥85% similarity were always used for further analyses. Only 2% of all strains tested had to be retested.

### Creating Superspectra corresponding to PFGE-types

**Fig. 1:**
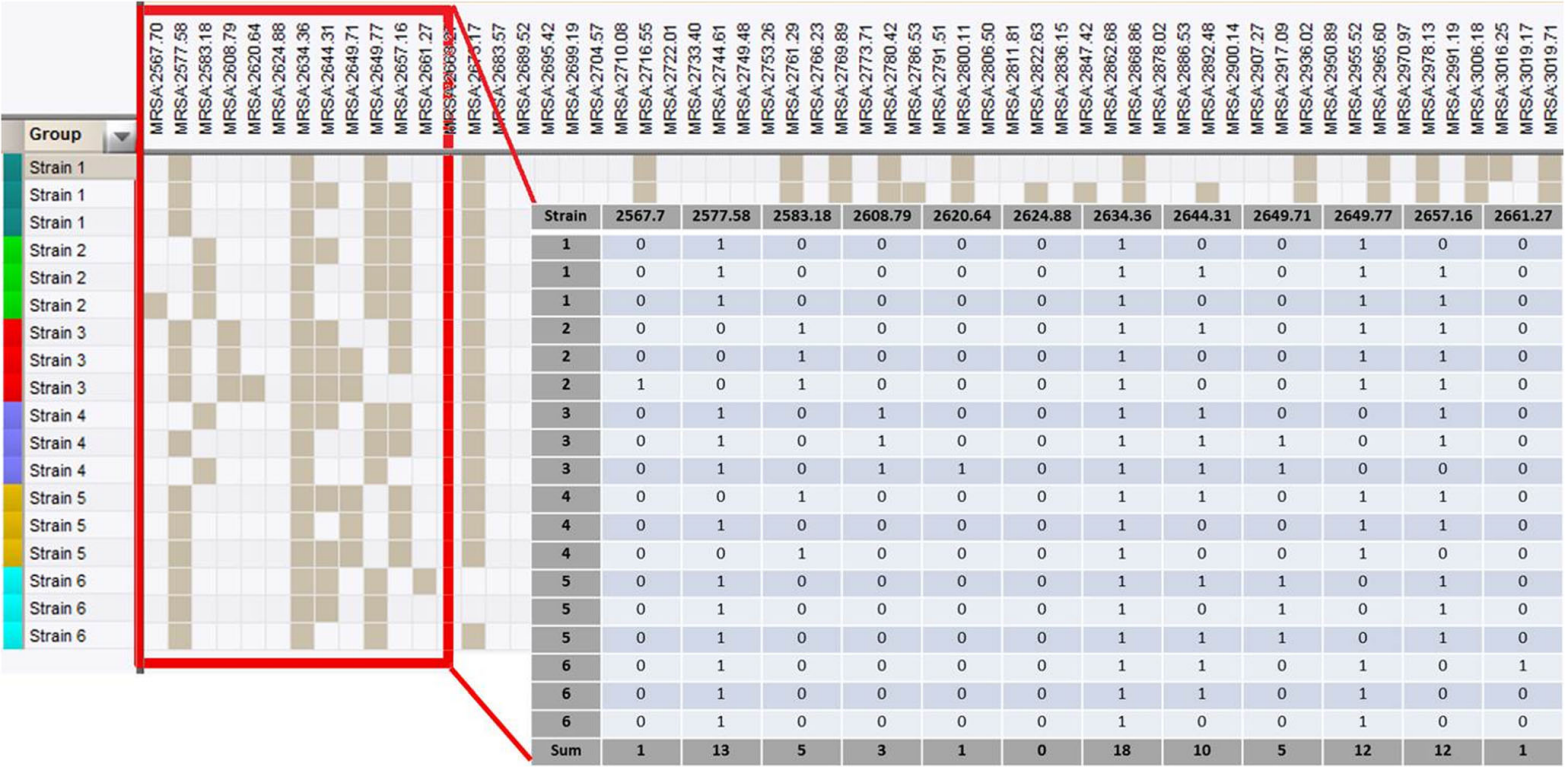
Selection of peaks to create Superspectra was performed by analysing presence or absence of particular peaks using the BioNumerics software. The frequency of peaks from a selection of 111 *Superspectra strains* belonging to certain PFGE-types was analysed both within the specific PFGE-types as well as within the whole *Superspectra strain* population. *Filled squares* indicate presence of a specific peak and *empty squares* indicate absence of this peak. The patterns of absent/present peaks were imported into an Excel sheet in which present peaks are designated 1 and absent designated 0 (as demonstrated by the magnified selection). The sum of each column (peak) indicates the frequency of a particular peak within the population studied (single PFGE-type or the whole *Superspectra strain* population)

MALDI-TOF MS peak lists of all 111 *Superspectra strains* were imported from SARAMIS into the BioNumerics version 7.5 software. The peak lists were compared, using the composite dataset function in BioNumerics, generating a pattern of present and absent peaks for each spectrum as shown in Fig. 1. No account was given for the intensity of the peaks. Peak matching was made with a tolerance of 0.08%. The peak patterns were imported into an Excel sheet in which present peaks were designated as ‘one’ and absent peaks as ‘zero’. The frequency of respective peaks could then be calculated by summing the peak columns individually. The peak frequencies within each PFGE-type of the *Superspectra strains*, as well as within the whole MRSA population studied, were calculated.

For each PFGE-type, ten peaks were selected for creation of a Superspectrum according to the following criteria: (i) high frequency of a given peak within the specific PFGE-type and (ii) low frequency within the whole population. If two peaks with identical frequency in the whole *Superspectra strain* population were identified as candidate peaks, the peak with the highest frequency in the PFGE-type was preferred.

Each Superspectrum consisted of ten peaks. To account for possible differences in specificities among the peaks, each peak was given a weight as described below. The total weights of the peaks in any given Superspectrum were summed to a value of 100. Three different strategies were evaluated for weighting the peaks: (i) all peaks were weighted equally, i.e. each peak received the same value, 10; (ii) the second strategy took into account how common a specific peak was within a given PFGE-type, as determined using the equations:$\frac{x}{y_{PFGE}}=z{\frac{x}{y_{PFGE}}}^{\ast }100= weig ht,\kern1em$where x = number of spectra containing that peak within the given PFGE-type, y_PFGE_ = total number of spectra within the PFGE-type, z_tot_ = all z-values within a Superspectrum; (iii) the third strategy included the frequency of peaks in the whole population, with the equations: $\kern0.75em {\frac{x}{y_{PFGE}}}^{\ast}\frac{x}{y_{PFGE}}=z{\frac{x}{y_{PFGE}}}^{\ast }100= weig ht$, where y_tot_ = total number of spectra containing that peak within the total MRSA population.

The result of the selection and weighting processes were then transferred to the VITEK MS RUO database, SARAMIS (bioMérieux), and using the Superspectra function creating PFGE-specific Superspectra. Superspectra were stored in a separate folder in the local SARAMIS database for further use in analysis.

### Statistical analysis

The Mann-Whitney test was used for comparison of similarity values of the first and second best matching Superspectrum of strains identified to the expected MALDI-type. The software GraphPad Prism 7 (GraphPad Software, La Jolla, CA, USA) was used for the calculations.

## Results

### Creating a database of Superspectra corresponding to PFGE-types

To use SARAMIS for sub-typing MRSA strains, a database using the *Superspectra strains* was created, consisting of 19 Superspectra representing the 19 most common MRSA PFGE-types in the region.

For each PFGE-type, ten MS peaks were selected, resulting in a combination of peaks in a Superspectrum, which is an artificial spectrum specific for that PFGE-type. The individual peaks comprising a Superspectrum were chosen according to their relative frequencies. For the selected peaks associated with a particular PFGE-type, the frequency of a given peak for strains of the respective PFGE-type varied from 73 to 91% (mean 81%, median 78%), whereas for the whole set of *Superspectra strains*, the frequencies were 18–42% (mean 31%, median 34%). Thus, selected peaks were present at varying frequencies within each PFGE-type and in the whole population (Supplementary information, Table S2). The peak at m/z 4457 was, for example, present in seven Superspectra while the peak at m/z 4184 was present in only one Superspectrum. The reproducibility of the selected peaks in the same isolate at separate occasions were found to be high (data not shown).

To compensate for the difference in specificity between peaks, three different weighting systems were developed and evaluated as described in the Materials and Methods section. The best results were obtained when both the frequencies within each PFGE-type and in the whole population were taken into account (the third approach). Peaks present in several Superspectra received a lower weight, compared to peaks present in strains of only one PFGE-type. This was selected as the final method for calculating the weights of the peaks in the Superspectra. The calculated weights of the peaks ranged from 3 to 32 (median value 8). In total, 115 different peaks were used for creating Superspectra given that, on average, each peak was present in 1.7 (range 1–7) of the 19 Superspectra (Supplementary information, Table S2).

### Determining threshold peak matching value

The matching of a strain’s spectra to the created Superspectra was, after fulfilling the initial criteria to generate three replicate spectra (all of which were fulfilling the 85% similarity criteria in the SARAMIS software as outlined in Material and Methods), always dependent on all three replicates. Each replicate spectrum was compared one by one to each Superspectrum in the database. The sum of the weights for each spectrum was calculated by adding the weights of the peaks in the spectrum matching the peaks in the Superspectrum (ranging from 0 to 100, which equals 0–100%). The peak matching values of the three replicates were added together to get the mean peak matching value (%) of the strain to a particular Superspectrum.

**Fig. 2:**
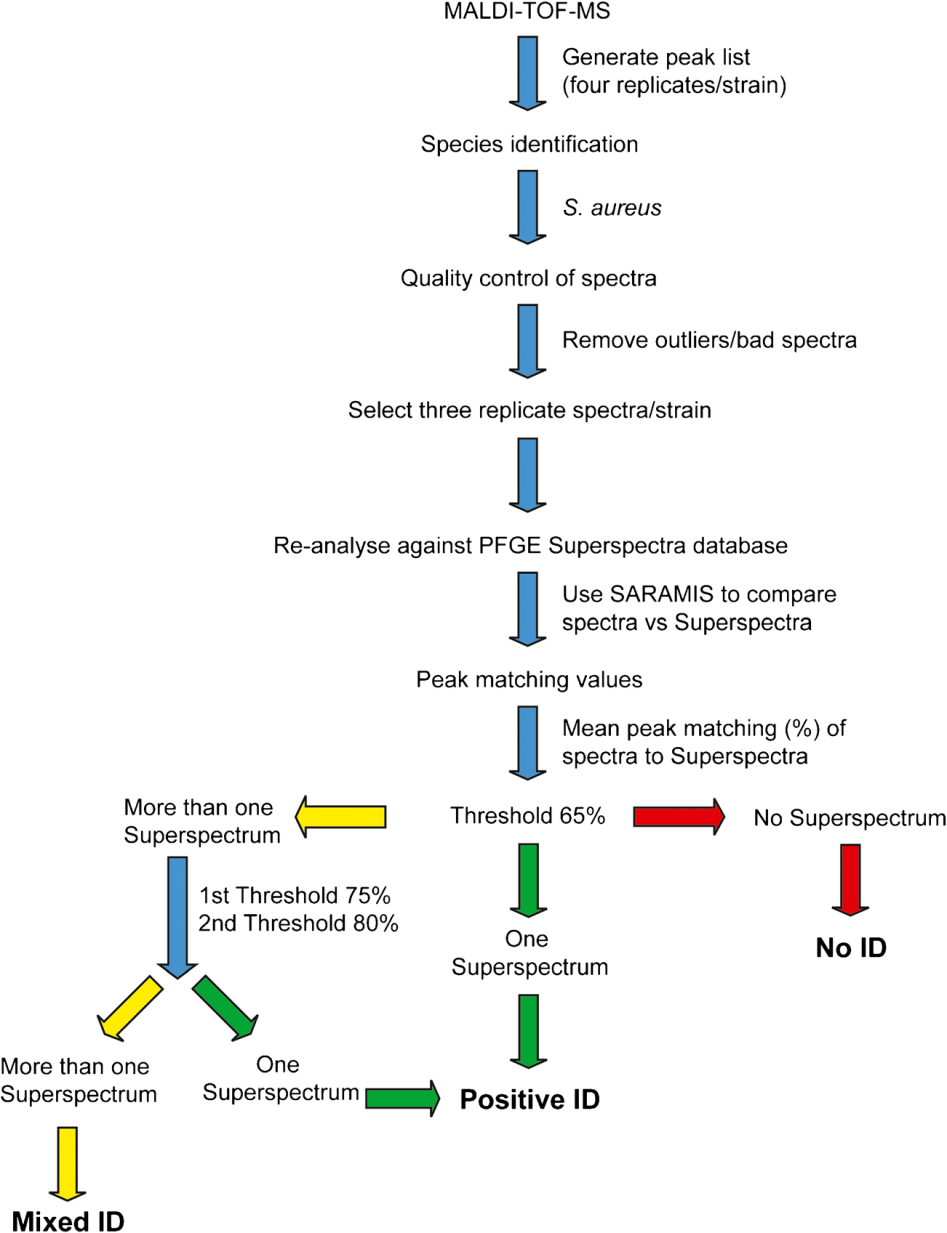
Workflow of the analysis of the spectra. Three replicate spectra generated with MALDI-TOF MS were selected for analysis of each strain. The spectra were compared to the PFGE-specific Superspectra database generated in this study and the number of matching peaks was compared to the 65%, 75% and 80% thresholds in order to assign the tested strain to a PFGE-specific Superspectra

The definition of a match to a Superspectrum was first set at choosing the Superspectrum with the highest mean peak matching value. However, this rendered a large number of inconsistent typing results, according to the known PFGE-types. Thus, different thresholds were evaluated using the test strains (data not shown). As a result of this evaluation the final analysis followed the procedure outlined in Fig. 2. If a strain matched only to a single type-specific Superspectrum in the database with a mean peak matching value of ≥65% the strain was considered to be “identified” as possibly belonging to this type. If the strain had a peak matching value ≥65% to several Superspectra the threshold was increased up to 80% (however ≥75% was the level generally needed to match a strain to a single Superspectra). If there were more than one possible match above the 80% threshold this was considered “Mixed identity” and if no peak matching to a single Superspectrum reached 65% this was considered “No identity” with respect to any of the 19 Superspectra or MALDI-types, respectively, in the database.

### Evaluation and modification of the Superspectra database

The accuracy of each Superspectrum using the thresholds as described in Fig. 2 was evaluated in relation to the known PFGE–types of the included *Superspectra strains* in two steps: first, it was compared to the *Superspectra strains* used to create it, of the same PFGE-type. Second, all *Superspectra strains*, regardless of PFGE-type, were analysed. If the Superspectrum failed to identify the strain to the correct PFGE-type or misidentified strains of other PFGE-types to the respective Superspectra, other combinations of peaks were evaluated to generate more specific Superspectra to the respective PFGE-types. The newly generated Superspectrum was then evaluated with the above described procedure and if better performance was achieved this Superspectrum was used as a PFGE-type specific Superspectrum in the following evaluation.

Not surprisingly, the specificity was not 100% to most Superspectra. Some of the Superspectra were cross-matching, resulting in “false matches” and mixed identifications. The 19 Superspectra, each representing one of the 19 PFGE-types, were therefore combined into ten groups, labelled MALDI-types, namely, M1–10 (Table 1). Grouping of the Superspectra that cross-matched were based only on MALDI-TOF MS characteristics disregarding other strain characteristics such as MLST and *spa*-type. Each MALDI-type represented strains belonging to one to five PFGE-types (Table 1). Five MALDI-types corresponded to a single PFGE-type (M1, M3, M6, M9 and M10). M2 and M8 each comprised two PFGE-types of the same MLST sequence type (ST) and *spa-*types. Two MALDI-types, M5 and M7, each consisted of several PFGE-types with strains of multiple ST or *spa-*types or both (Tables 1 and 2). The five PFGE-specific Superspectra that were included in M7 consisted of 30 peaks (Supplementary information Table S2), of which 18 were present in only one Superspectrum and only two of the peaks were shared among all five Superspectra.

In the database the original PFGE-type specific Superspectra were kept and the designation of a strain to a MALDI-type was based on matching of the strain to a PFGE-specific Superspectrum within that MALDI-type.

### Evaluation of Superspectra performance with test strains

**Fig. 3:**
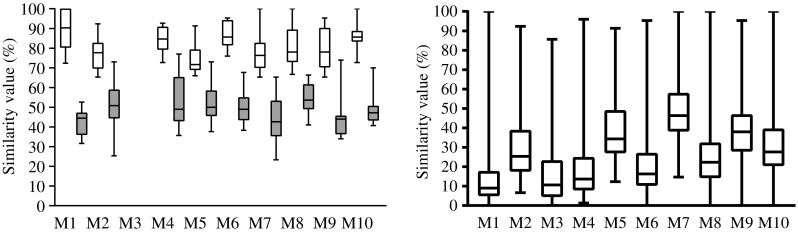
**a** *Test strains* that were identified to the expected MALDI-type according to PFGE-type (*n* = 160). The figure shows the difference of peak matching values (%) between the first and second best matching MALDI-types. Statistics were calculated with the Mann-Whitney test. **b** Peak matching value of all analysed *test strains* to all MALDI-types

All replicates of the 255 MRSA *test strains* were first verified to be *S. aureus* with SARAMIS using standard procedures. As a second step the stored replicate spectra were reanalysed one at a time against the 19 PFGE-type Superspectra in the database and classified into the ten MALDI-types. In total, 172 strains (67.4%) were assigned to the correct MALDI-types, including 12 strains belonging to a PFGE-type not represented in the database that—correctly—did not yield matches to any Superspectrum (Table 3). The median value for correct assignment to a MALDI-type was 78% (range 65.3–100%), whereas the second-best matching Superspectra had a significantly lower value, with a median of 49% (range 23.3–77.0%, *p* < 0.0001; Fig. 3a and b). Re-analysis of isolates at different occasions corresponded well; no conflicting results were observed (data not shown).

**Table 3:** Results of MALDI-typing with Superspectra of 255 independent *test strains*. MALDI-types M1-M10 refers to groups of PFGE-types as given in Table 1. Reference typing results for the set of strains is given in Table 2

MALDI-type	Total number of strains	Correct ID^a^		Mix^b^		No ID^c^		Wrong ID^d^	
		Number, *n*	Percent, %	Number, *n*	Percent, %	Number, *n*	Percent, %	Number, *n*	Percent, %
M1	15	12	80,0	0	0	2	13,3	1	6,7
M2	37	24	64,9	0	0	11	29,7	2	5,4
M3	1	0	0,0	0	0	1	100	0	0,0
M4	19	15	78,9	1	5,3	2	10,5	1	5,3
M5	41	19	46,3	1	2,4	17	41,5	4	9,8
M6	16	13	81,3	0	0,0	3	18,8	0	0,0
M7	41	28	68,3	2	4,9	11	26,8	0	0,0
M8	30	20	66,7	0	0,0	9	30,0	1	3,3
M9	14	11	78,6	0	0,0	3	21,4	0	0,0
M10	24	18	75,0	1	4,2	4	16,7	1	4,2
Other^e^	17	12	70,6	1	5,9	0	0,0	4	23,5
Total	255	172	67,4	6	2,4	63	24,7	14	5,4

^a^Correct ID; Assignment to expected MALDI-type

^b^Mix; Match to two or more MALDI-types

^c^No ID; No assignment above the 65% peak matching threshold

^d^Wrong ID; Assigned to a different MALDI-type than the expected with respect to PFGE-type

^e^Others refer to strains with PFGE-types for which no Superspectrum were present in the database

For 39 of the 255 (15.3%) *test strains* the mean peak matching value to the expected Superspectrum was 61.7% (range 45.3–64.7%) indicating possible similarity, but still below the threshold of 65%, and for 24 strains (9.4%) the best matching Superspectrum/MALDI-type were incorrect with regard to PFGE-type. Therefore, these strains could not be assigned to any of the ten MALDI-types, although the expected MALDI-type was present in the database. Only 14 (5.5%) of the strains were incorrectly assigned to another MALDI-type than the expected based on the PFGE-type of the strain (Table 4). Four of these 14 strains were of a PFGE-type not represented among the *Superspectra *strains and did thus not have a corresponding Superspectrum. An additional three strains were incorrectly assigned to MALDI-types containing strains with that same *spa*-type, while the PFGE-type was different and another two strains were assigned to MALDI-types (M1 and M4) that shared MLST and *spa*-types.

**Table 4:** Incorrectly assigned and mixed ID strains according to MALDI-type/PFGE-type

ID	Peak matching value	Expected MALDI-type	Peak matching value of expected MALDI-type	*spa-*CC *(spa*-type)	PFGE-type	Comment
M7	68.3%	n.a.	–	345/359 (t267)	–	No Superspectra available in database
M3	85.6%	n.a.	–	t991^a^	–	No Superspectra available in database
M3	68.3%	n.a.	–	t991^a^	–	No Superspectra available in database
M7	78.7%	n.a.	–	008 (t008)	–	No Superspectra available in database
M4	87.7%	M1	16.3%	012 (t021)	B	The *spa*-type of the strain correlates with the most common *spa*-type in the matching MALDI-type
M1	75%	M4	38.6%	012 (t019)	SM	The *spa*-type of the strain correlates with the most common *spa*-type in the matching MALDI-type
M7	81.3%	M5	16%	002 (t002)	R	The *spa*-type of the strain correlates with the most common *spa*-type in the matching MALDI-type
M7	75.3%	M5	15%	008 (t304)	R	The *spa*-type of the strain is common in both M5 and M7
M7	78.7%	M5	60.7%	008 (t304)	R	The *spa*-type of the strain is common in both M5 and M7
M5	79%	M10	14%	–	T	
M5	68.7%	M2	42.3%	t272^b^	I	
M7	83%	M2	78.7%	790/005 (t790)	BK	
M9	65%	M5	57%	t127^b^	R	
M7	75.7%	M8	17.7%	008 (t1476)	K	
M7/M9	71/73.7%	M7	71%	345/359 (t657)	J	Mixed ID
M7/M10	65/66%	M7	65%	345/359 (t1839)	J	Mixed ID
M5/M9	68.3/69%	n.a.	–	091 (t084)	–	Mixed ID, no Superspectra available in database
M5/M7	65.3/65%	M5	65%	008 (t701)	R	Mixed ID
M4/M5	91.3/86.7%	M4	91.3%	012 (t012)	SM	Mixed ID
M9/M10	80.3/97%	M10	97%	448/690 (t690)	T	Mixed ID

^a^Excluded from cluster analysis due to less than four repeats

^b^Singletons

## Discussion

Epidemiological surveillance and typing of bacterial strains is important to determine the source of the infection and for preventing the transmission of infectious agents. In an outbreak situation, turnaround time can be of utmost importance. MALDI-TOF MS is a rapid and inexpensive method that may prove to serve an important role as a screening tool for rapid epidemiological typing and tracking of outbreaks, especially in a local setting.

This study presents a simple and fast method for epidemiological screening suitable for a large number of samples. The proposed value of this method is as a first step in a typing scheme of MRSA isolates, mainly to exclude relationship in a chain of possible transmission rather than to do exact strain sub-typing. The results could be used to urgently determine the need of initiating an outbreak investigation with further typing or not at all. If strain relatedness is suspected, more in-depth molecular typing methods are more suitable to determine the clonality of the isolates [18–20]. MALDI-TOF MS typing may also function as a quick complementary test when more conventional tests like *spa*-typing give inconclusive results.

In this study we have compared the MALDI-TOF MS typing primarily to PFGE-typing data of the MRSA strains. To compare the Superspectra database with *spa*-type or MLST would have based the method on more objective grounds. On the other hand, studies have shown that PFGE-typing is a discriminatory typing method in an outbreak situation [21]. The *Superspectra strains* were chosen as representative strains of the most common PFGE-types in the region of South-West Sweden and several subtypes of 70% identity or more within the respective PFGE type were included as well.

Other attempts have been made to establish MALDI-TOF MS-based screening methods for MRSA [14–16, 22, 23]. However, of the 115 peaks used to construct our Superspectra database, only 12 peaks are shared with these other studies. The specificity of our approach is further ensured since the combination of peaks and their individual relative contribution, reflected by different weights, is considered for each Superspectrum. This also allows particular peaks to be present in more than one Superspectrum and thus the number of studied peaks can be enlarged. Biomarkers presumed to be specific for given subsets of strains often lose their specificities when more strains are introduced into the analyses [24]. Furthermore, as argued by Lasch et al. [25], it is unlikely that single-marker peaks will be sufficiently discriminatory to identify a given subset of strains.

In this study no effort has been made to identify the corresponding proteins of the peaks or the functions thereof, hence this method could be set up for any given microorganism without any in-depth information regarding the identity of the proteins. However, the risk of not knowing the identity of the proteins include introduction of non-specific peaks, originating from, for example, the growth media. In this study growth media specific peaks were excluded by applying an exclusion list automatically removing horse blood agar specific peaks.

We have constructed the PFGE-specific Superspectra in a similar way that the Superspectra of VITEK MS RUO/SARAMIS is constructed. As for any database, the coverage depends on how generally applicable the data it is built on is. The database presented in this study is only tested using strains from a limited region. In its current form it may only be applicable to this local setting. To use the method in other settings, strain types present in those localities need to be included and compared to the types of strain in the current database. A database such as this one also needs to be adjusted over time. We used historical data to identify the most common PFGE-types in the region for the past 20 years when constructing our Superspectra. When evaluating the database, some of the PFGE-types for which a Superspectrum was created were less common or absent among the more recently isolated *test strains*. On the other hand this demonstrates the specificity of our protocol.

Low discriminatory power has serious implications as this may hamper or delay discovery of an outbreak. A combination of tests has been suggested to increase the discriminatory power [21]. For instance, lack of discriminatory capacity for *spa*-typing within *spa-*type t304 is well known; several PFGE-types have been attributed to this *spa*-type [26, 27]. In concordance with those studies the 11 strains with this *spa*-type (illustrated as *spa*-CC 008, MLST-CC8 in Tables 1 and 2) included in our study were classified as belonging to different PFGE-types. As only five out of the 11 strains were correctly assigned to the expected MALDI-type with respect to PFGE-type, basing the MALDI-type on *spa*-type rather than PFGE-type would probably in this case have resulted in a more correct assignment since PFGE-typing is most useful for outbreak investigations and not for comparisons over time. If the typing is based on *spa*-type rather than PFGE, “difficult” *spa*-types such as t304 would then immediately be subject to further typing with, for example, NGS to determine clonality. In addition, *spa*-typing has the advantage of better reproducibility between different laboratories and the nomenclature is uniform in contrast to PFGE, and *spa*-types can more easily be compared worldwide.

In this study, only 5.5% of the *test strains* were mistyped, i.e. assigned to a different MALDI-type than expected (Table 4). Four of these strains were of a PFGE-type not represented among the *Superspectra *strains and thus did not have a corresponding Superspectrum. Possibly, by including these PFGE-types to form additional Superspectra in the database, this problem could have been solved. It should however be kept in mind that mistyping of MRSA strains using MALDI-typing does not automatically lead to final mistyping of the isolate. MALDI-typing is only to be used as a first step in the typing scheme and the clinical background of the samples in an outbreak situation should always be taken into account when the urge for further typing is considered.

Combining the Superspectra into MALDI-types was only based on cross-matching of the Superspectra. However, it was noticed that several groups of Superspectra that were difficult to separate represented strains belonging to the same ST by MLST, for example, the strains of PFGE-types K and M all belonging to ST8 were often cross-matched (Table 1).

There have been several studies using MALDI-TOF MS for sub-species typing of several different bacterial species, such as *Listeria monocytogenes* [28], *Acinetobacter spp.* [29] *Propionibacterium acnes* [30], *Clostridium difficile* [4] and MRSA [14, 16]. In general, MALDI-TOF MS is recognised to be effective for species-level identification but may lack the resolution that can be obtained by molecular-based methods for reliable sub-species analyses. Using standard settings, MALDI-TOF MS is limited in its ability to differentiate closely-related organisms, and special handling of the sample or other algorithms for comparison are often required. Protocols required for sub-species level typing are dependent on the features that are being investigated and need to be optimised for each particular taxon. However, a great advantage of the methodology presented in this paper is the use of standard settings for analysis, without prior preparations, such as ethanol or acetonitrile extraction. This makes the procedure easier for the routine clinical laboratory to apply on large numbers of samples. Also, the analysis of the data is straightforward; there is no need for interpretations of data as there are fixed thresholds set for the analysis in much the same way as the VITEK MS IVD is functioning. Even though setting the threshold is somewhat subjective, once set, the threshold is fixed for all analysed strains. Furthermore, the MALDI-typing is only a first step of typing and can be compared between laboratories if the databases are shared.

In order to use MALDI-typing for more in-depth typing means that the resolution would have to be increased. More extensive sample preparations have been shown to increase the resolution and enable typing [5, 6]; however, this means that one of the major advantages of whole-cell MALDI-TOF MS, the rapid turnaround time from sample to result, would be lost.

PFGE and *spa*-typing are methods capable of detecting substantial intra-species diversity to a degree that MALDI-typing may not [31]. However, these methods are both labour intensive and costly and while results of *spa*-typing and to some degree MALDI-typing can easily be shared between laboratories, PFGE results cannot. PFGE may be useful in outbreak situations because it can differentiate strains with minor genetic changes; however, both PFGE and other sequence-based methods have been shown to suggest close relationship between MRSA isolates while they actually differ when it comes to pathogenesis [32]. Therefore, we suggest the use of MALDI-typing for a rapid screening and when more in-depth typing is prompted for, NGS-based methods should be used to get the most accurate epidemiological typing.

The MALDI-TOF MS-based typing in this study has been developed using MRSA as a model system and based on PFGE-data. However, the method for building and using the database could be applied to other microorganisms in need of inexpensive real-time epidemiological surveillance.

To conclude, in this study we have developed and applied a method for rapid epidemiological screening of MRSA, using VITEK MS RUO/SARAMIS. The method is fast and cost-efficient and could be a valuable screening tool in epidemiological typing.

## Electronic supplementary material

ESM 1 (DOCX 104 kb)
