# Supplementary material for: Development of a rapid MALDI-TOF MS based epidemiological screening method using MRSA as a model organism
Source: Eur J Clin Microbiol Infect Dis. 2017 Sep 18;37(1):57–68. doi: 10.1007/s10096-017-3101-x (PMC5748427; doi:10.1007/s10096-017-3101-x)
Supplement: Supplementary file 1 — (DOCX 104 kb) [file 10096_2017_3101_MOESM1_ESM.docx]

Supplementary data

**Journal:** European Journal of Clinical Microbiology and Infectious Diseases

**Title:** Development of a rapid MALDI-TOF MS based epidemiological screening method using MRSA as a model organism

**Authors:** Åsa Lindgren^1#^, Nahid Karami^1,3^, Roger Karlsson^1,2,3^, Christina Åhrén^1,3,4^, Martin Welker^5^, Edward R.B. Moore^1,3,6^, Liselott Svensson Stadler^1^

**Affilations:**

^1^ Department of Infectious Diseases, Institute of Biomedicine, Sahlgrenska Academy of the University of Gothenburg, Gothenburg, Sweden

^2^ Nanoxis Consulting AB, Gothenburg, Sweden

^3^ Centre for Antibiotic Resistance Research (CARe) at University of Gothenburg, Gothenburg, Sweden

^4^ Swedish strategic programme against antibiotic resistance, Region Västra Götaland, Gothenburg, Sweden.

^5^ bioMérieux SA, Unit Microbiology, R&D Microbiology, La Balme Les Grottes, France.

^6^ Culture Collection University of Gothenburg (CCUG), Gothenburg, Sweden

**Corresponding author**; # e-mail: asa.lindgren@microbio.gu.se

**Table S1:** Strains included in study.

| **CCUG number** | ***spa-***type | **MALDI-type** | **CCUG number** | ***spa-*type** | **MALDI-type** | **CCUG number** | ***spa-*type** | **MALDI-type** | **CCUG number** | ***spa-type*** | **MALDI-type** |
| --- | --- | --- | --- | --- | --- | --- | --- | --- | --- | --- | --- |
| 27050 | t002 | M7 | 58656 | t685 | M4 | 66674 | t306 | M7 | 67007 | t325 | M10 |
| 33115 | t044 | M9 | 58954 | t223 | M2 | 66682 | t309 | M2 | 67017 | t3196 | M3 |
| 33735 | t018 | M4 | 59083 |  | M10 | 66685 | t690 | M10 | 67029 | t15556 |  |
| 35478 | t012 | M4 | 59097 | t019 | M1 | 66686 | t223 | M2 | 67030 | t304 | M5 |
| 35695 | t012 | M4 | 59241 | t685 | M4 | 66687 | t5041 | M10 | 67031 | t019 | M1 |
| 36596 | t012 | M4 | 59681 | t008 | M8 | 66688 | t10682 | M7 | 67032 | t314 |  |
| 37315 | t748 | M4 | 59721 | t021 | M4 | 66689 | t325 | M10 | 67033 | t306 | M7 |
| 38266 |  | M4 | 59874 | t5773 | M5 | 66693 | t019 | M1 | 67039 | t223 | M2 |
| 38979 | t1233 | M2 | 60135 | t064 | M8 | 66694 | t127 | M5 | 67040 | t386 |  |
| 39530 | t172 | M5 | 60209 | t437 | M5 | 66695 | t002 | M7 | 67041 | t002 | M7 |
| 41675 | t002 | M7 | 60293 | t008 | M8 | 66697 | t954 | M7 | 67042 | t701 | M5 |
| 41688 | t214 | M7 | 60393 | t223 | M2 | 66703 | t044 | M9 | 67053 | t044 | M9 |
| 41764 | t127 | M5 | 60426 | t324 | M3 | 66704 | t084 |  | 67054 | t008 | M8 |
| 41787 | t050 | M6 | 60428 | t690 | M10 | 66705 | Non typeable |  | 67055 | t304 | M5 |
| 42348 | t015 | M6 | 60469 | t7358 | M7 | 66706 | t690 | M10 | 67067 | t008 | M8 |
| 42822 | t032 | M2 | 60531 | t690 | M10 | 66709 | t690 | M10 | 67073 | t002 | M7 |
| 43063 | t2363 | M6 | 60550 | t1839 | M7 | 66710 | t11550 | M10 | 67082 | t375 |  |
| 43232 | t044 | M9 | 60640 | t852 | M2 | 66819 | t008 | M8 | 67083 | t019 | M1 |
| 43826 | t019 | M1 | 60712 | t5981 | M7 | 66821 | t690 | M10 | 67095 | t034 |  |
| 43863 | t002 | M7 | 60862 | t018 | M4 | 66822 | t5100 | M5 | 67096 |  | M5 |
| 43895 | t267 |  | 60993 | t8503 | M7 | 66823 | t044 | M9 | 67098 |  | M5 |
| 44309 | t1021 | M10 | 61069 |  | M1 | 66828 | t230 | M6 | 67099 | t325 | M10 |
| 44410 | t044 | M9 | 61196 | t010 | M7 | 66829 | t005 | M2 | 67115 | t1062 | M7 |
| 44464 | t008 | M3 | 61281 | t163 | M5 | 66830 | t304 | M5 | 67123 | t071 | M7 |
| 44884 | t044 | M9 | 61351 | t790 | M2 | 66831 | t008 | M8 | 67129 | t127 | M5 |
| 44953 |  | M10 | 61494 | t664 | M3 | 66852 | t127 | M5 | 67130 | t002 | M7 |
| 45435 | t012 | M4 | 61734 |  | M7 | 66853 | t223 | M2 | 67148 | t1476 | M8 |
| 45555 | t015 | M6 | 61745 | t2315 | M7 | 66860 | t019 | M1 | 67149 | t008 | M8 |
| 45736 | t313 | M4 | 61793 | t6717 | M2 | 66861 | t019 | M1 | 67150 | t002 | M7 |
| 46140 | t021 | M4 | 61825 | t9697 | M10 | 66862 | t019 | M1 | 67151 | t044 | M9 |
| 46314 | t002 | M7 | 61902 | t6750 | M5 | 66873 | t008 | M8 | 67161 | t127 | M5 |
| 46315 | t044 | M9 | 62184 | t2251 | M2 | 66874 | t008 | M8 | 67171 | t690 | M10 |
| 46755 |  | M10 | 62228 | t7387 | M2 | 66875 | t304 | M5 | 67172 | t690 | M10 |
| 47432 |  | M7 | 62256 | t5841 | M9 | 66890 | t304 | M5 | 67187 | t1784 | M5 |
| 47506 | t044 | M9 | 62258 |  | M8 | 66891 | t954 | M7 | 67188 | t1784 | M5 |
| 47605 | t008 | M8 | 62518 | t019 | M1 | 66892 | t002 | M7 | 67189 | t019 | M1 |
| 47719 | t008 | M8 | 62593 | t223 | M2 | 66902 | t7284 | M7 | 67190 | t002 | M5 |
| 48111 | t008 | M8 | 62624 | t448 | M10 | 66903 | t127 | M5 | 67200 | t005 | M2 |
| 48265 | t019 | M4 | 62740 | t024 | M8 | 66904 | t044 | M9 | 67201 | t334 | M8 |
| 49035 | t437 | M5 | 62806 | t019 | M1 | 66905 | t370 | M6 | 67203 | t690 | M10 |
| 49487 | t127 | M5 | 62904 | t657 | M7 | 66906 | t019 | M1 | 67204 | t304 | M7 |
| 49617 | t630 | M6 | 62915 | t127 | M5 | 66907 | t008 | M8 | 67205 | t790 | M2 |
| 49631 |  | M9 | 62939 | t1752 | M1 | 66908 | t019 | M1 | 67206 | t044 | M9 |
| 49663 | t015 | M6 | 63012 | t127 | M5 | 66909 | t304 | M5 | 67207 | t085 |  |
| 49785 |  | M5 | 63366 | t1774 | M8 | 66911 | t441 | M5 | 67211 | t304 | M5 |
| 50648 | t018 | M4 | 63429 | t991 |  | 66914 | t005 | M2 | 67212 | t7284 | M7 |
| 51264 | t127 | M5 | 63677 | t334 | M8 | 66915 | t437 | M5 | 67223 | t437 | M5 |
| 51345 | t044 | M9 | 63776 | t11903 | M7 | 66916 | t019 | M4 | 67243 | t127 | M5 |
| 51451 | t1178 | M5 | 63797 | t4892 | M2 | 66921 | t2396 | M7 | 67249 | t304 | M5 |
| 51888 | t306 | M7 | 63827 | t535 | M7 | 66922 | t005 | M2 | 67250 | t223 | M2 |
| 52258 | t127 | M5 | 64007 | t105 | M7 | 66923 | t127 | M5 | 67251 | t648 | M7 |
| 52348 | t102 | M6 | 64039 | t437 | M5 | 66924 | t005 | M2 | 67254 |  | M7 |
| 52349 | t102 | M6 | 64126 | t345 | M7 | 66925 | t331 | M6 | 67255 | t127 | M5 |
| 52386 | t421 | M4 | 64303 | t021 | M4 | 66926 | t272 | M2 | 67256 | t657 | M7 |
| 52506 | t015 | M6 | 64323 | t019 | M1 | 66927 | t019 | M1 | 67263 | t026 | M6 |
| 52535 | t008 | M8 | 64377 | t008 | M8 | 66928 | t008 | M8 | 67264 |  | M7 |
| 52774 | t018 | M4 | 64531 | t13115 | M8 | 66929 | t044 | M9 | 67283 | t044 | M9 |
| 52859 | t390 | M6 | 64558 | t127 | M5 | 66930 | t223 | M2 | 67284 |  | M7 |
| 52915 | t583 | M6 | 64560 | t4623 | M2 | 66931 | t10892 | M9 | 67285 | t002 | M7 |
| 53041 | t015 | M6 | 64564 | t570 | M7 | 66932 | t991 |  | 67286 | t223 | M2 |
| 53053 | t015 | M6 | 64565 | t005 | M2 | 66933 | t008 | M8 | 67287 | t504 | M5 |
| 53135 | t050 | M6 | 64580 | t018 | M4 | 66936 | t008 | M8 | 67288 | t2649 | M10 |
| 53315 | t008 | M8 | 64689 | t008 | M8 | 66944 | t701 | M5 | 67289 |  | M10 |
| 53525 | t015 | M6 | 64692 | t3876 | M7 | 66945 | t105 | M7 | 67290 | t024 | M8 |
| 53542 | t002 | M7 | 64697 | t223 | M2 | 66946 | t021 | M4 | 67291 | t067 | M7 |
| 53606 | t950 | M6 | 64894 | t688 | M7 | 66947 | t085 | M4 | 67292 | t1504 |  |
| 53826 | t131 | M9 | 65019 | t701 | M5 | 66949 | t008 |  | 67293 | t690 | M10 |
| 54382 | t318 | M4 | 65190 |  | M2 | 66950 | t304 | M5 | 67294 |  | M2 |
| 54462 | t026 | M6 | 65380 | t4145 | M5 | 66951 | t004 | M9 | 67308 | t9987 | M7 |
| 54488 | t416 | M9 | 65474 | t324 | M3 | 66952 | t223 | M2 | 67309 | t345 | M7 |
| 54495 | t2364 | M4 | 65623 | t345 | M7 | 66956 | t005 | M2 | 67313 | t14986 | M10 |
| 54671 | t044 | M9 | 65648 | t6662 | M7 | 66961 | t002 | M7 | 67318 | t086 | M10 |
| 54828 | t390 | M6 | 65719 | t1476 | M8 | 66962 | t4407 | M5 | 67323 | t790 | M2 |
| 54868 | t400 | M8 | 65755 | t091 | M5 | 66963 | t127 | M5 | 67324 | t3507 | M2 |
| 55198 | t012 | M4 | 65833 | t008 | M8 | 66964 | t202 | M8 | 67333 | t690 | M10 |
| 55260 | t012 | M4 | 65944 | t012 | M4 | 66966 | t786 | M10 | 67334 | t034 |  |
| 55415 | t102 | M6 | 65955 | t1784 | M5 | 66967 | t304 | M5 | 67335 | t223 | M2 |
| 55482 | t657 | M7 | 65970 | t211 | M8 | 66970 | t127 | M5 | 67336 | t021 | M4 |
| 55603 | t630 | M6 | 65975 | t148 | M3 | 66977 | t223 | M2 | 67337 | t2649 | M10 |
| 56171 |  | M2 | 65986 | t936 | M7 | 66978 |  | M7 | 67338 | t005 | M2 |
| 56592 | t223 | M2 | 66102 | t359 |  | 66979 | t690 | M10 | 67339 | t005 | M2 |
| 57043 | t190 | M8 | 66146 | t223 | M2 | 66980 | t690 | M10 | 67340 | t005 | M2 |
| 57152 | t4412 | M2 | 66211 | t019 | M1 | 66983 | t008 | M8 | 67341 | t4892 | M2 |
| 57185 | t064 | M8 | 66275 | t223 | M2 | 66984 | t688 | M7 | 67342 | t304 | M5 |
| 57215 | t024 | M8 | 66277 | t223 | M2 | 66993 | t105 | M7 | 67343 | t777 | M7 |
| 57295 | t064 | M8 | 66335 | t852 | M2 | 66994 | t105 | M7 | 67348 | t223 | M2 |
| 57568 | t685 | M4 | 66350 | t657 | M7 | 66996 | t044 | M9 | 67349 | t10239 | M8 |
| 57774 | t018 | M4 | 66385 | t002 | M7 | 66997 | t019 | M1 | 67350 | t122 | M1 |
| 57916 | t088 | M7 | 66401 | t304 | M5 | 66998 | t008 | M8 | 67351 | t044 | M9 |
| 58249 | t2910 | M4 | 66451 | t437 | M5 | 67004 | t1855 | M10 | 67352 | t664 | M3 |
| 58529 | t024 | M8 | 66462 | t267 |  | 67005 | t5071 |  |  |  |  |
| 58530 | t657 | M7 | 66546 | t172 | M5 | 67006 | t002 | M7 |  |  |  |

**Table S2**. Peaks used to construct the Superspectra. The letter x shows the presence of peak in the PFGE type. In parentheses are the weights of respective peak, weights above 15 are in bold letters. Weights calculated by method iii as stated in the Material and Methods section.

|  | M1 | M2 | | M3 | M4 | | M5 | | | M6 | M7 | | | | | M8 | | M9 | M10 |
| --- | --- | --- | --- | --- | --- | --- | --- | --- | --- | --- | --- | --- | --- | --- | --- | --- | --- | --- | --- |
| m/z | B | BK | I | C | Cy | SM | D | J2 | R | F | J3 | J | L | S | A | K | M | P | T |
| 2029 |  |  |  |  |  |  |  |  |  |  |  |  |  | x(8) |  |  |  |  |  |
| 2037 |  | x(3) |  |  |  |  |  |  |  |  |  |  | x(6) |  | x(6) |  |  |  |  |
| 2126 |  |  |  |  |  |  | x(10) |  |  |  | x(8) |  |  |  |  |  |  |  |  |
| 2174 |  |  |  |  |  |  | x(11) |  |  |  |  |  |  |  |  |  |  |  |  |
| 2198 |  |  | x(6) |  |  |  |  | x(7) |  |  |  |  |  |  |  |  |  |  |  |
| 2228 |  |  |  |  |  |  |  |  |  |  |  |  |  |  |  |  | x(9) |  |  |
| 2237 |  |  |  |  |  |  | x(6) |  |  |  |  |  |  |  |  |  |  |  |  |
| 2243 |  |  |  |  |  | x(7) |  |  |  |  |  |  |  |  |  |  |  |  |  |
| 2250 |  |  |  | x(5) |  |  |  | **x(21)** |  |  |  |  |  |  |  |  |  |  |  |
| 2256 |  |  |  |  | x(11) | x(8) |  |  |  |  |  |  |  |  |  |  |  |  |  |
| 2279 |  |  |  |  |  |  |  |  |  |  |  | x(5) | x(6) | x(7) |  |  | x(5) |  |  |
| 2321 |  |  |  |  |  |  |  |  |  |  |  |  | x(9) |  |  |  |  |  |  |
| 2388 |  |  |  |  |  |  |  | x(9) |  |  |  |  |  | x(7) |  |  | x(5) |  |  |
| 2407 |  |  |  |  |  | x(7) |  | x(8) |  |  |  |  |  |  |  |  |  |  |  |
| 2413 |  |  |  |  | **x(15)** |  |  |  |  |  |  |  |  |  |  |  |  |  |  |
| 2512 |  |  | x(13) |  |  |  |  |  |  |  |  |  |  |  |  |  |  |  |  |
| 2521 |  |  |  | x(4) |  |  |  |  |  |  | x(7) |  |  |  |  |  |  |  |  |
| 2549 |  |  |  |  |  |  |  |  | x(10) |  |  |  |  |  |  |  |  |  |  |
| 2577 |  |  |  |  |  |  |  |  |  |  |  |  |  |  |  |  |  | x(7) |  |
| 2583 |  |  |  |  |  |  |  |  |  |  | x(8) | x(9) | x(7) | x(12) |  |  |  |  |  |
| 2608 |  |  |  |  |  |  |  |  |  | x(10) |  |  |  |  |  |  |  |  |  |
| 2644 |  |  | x(7) |  |  |  | x(7) |  |  |  |  |  |  |  |  |  |  |  |  |
| 2673 |  |  |  |  |  |  |  |  |  |  |  |  |  |  |  |  |  |  | x(4) |
| 2710 |  |  |  |  | x(6) |  | x(6) |  |  |  |  |  |  |  |  |  |  |  |  |
| 2716 |  |  |  |  |  |  |  |  |  |  |  |  |  | x(12) |  |  |  |  |  |
| 2722 |  |  |  | x(12) |  |  |  |  |  |  |  |  |  |  |  |  |  |  |  |
| 2753 |  |  |  |  | x(4) |  |  |  |  |  |  |  |  |  |  |  |  |  |  |
| 2786 |  |  |  | x(10) |  |  |  |  |  |  |  |  |  |  |  |  |  |  |  |
| 2806 |  |  |  |  |  |  |  |  |  |  |  |  | x(9) |  | x(9) |  |  |  |  |
| 2847 |  |  |  |  |  |  |  |  |  |  | x(16) |  |  |  |  |  | x(5) |  |  |
| 2868 |  |  |  |  |  |  |  |  |  |  |  |  |  |  |  |  |  |  | x(4) |
| 2878 |  |  |  |  |  |  |  |  |  |  | x(11) | x(5) |  |  |  |  |  |  |  |
| 2892 |  |  |  |  |  |  |  |  |  |  |  | x(6) |  |  |  |  |  |  |  |
| 2907 |  |  |  |  |  |  | **x(17)** | x(12) |  |  |  |  |  |  |  |  |  |  |  |
| 2917 | x(10) |  |  |  |  |  |  |  |  |  |  |  |  |  |  |  |  |  |  |
| 2936 |  |  |  | x(5) | x(4) |  |  |  |  |  |  |  |  |  |  |  |  |  |  |
| 3022 |  |  |  |  |  |  |  |  |  |  |  |  | x(12) |  |  |  |  |  |  |
| 3028 | x(3) |  |  |  |  |  |  |  |  |  |  |  |  |  |  |  |  |  |  |
| 3035 |  |  |  |  |  |  | x(11) | x(8) |  |  |  | x(6) |  |  |  |  |  |  |  |
| 3054 |  |  |  |  |  |  | x(7) |  |  |  |  |  | x(9) |  | x(9) |  |  |  |  |
| 3066 |  | x(5) |  |  |  |  |  |  |  |  |  |  |  |  |  |  |  |  |  |
| 3074 |  |  |  |  |  |  | **x(15)** | x(9) |  |  |  |  |  |  |  |  |  |  |  |
| 3240 |  |  |  | x(4) |  |  |  | x(8) |  |  |  |  |  |  |  |  |  |  |  |
| 3295 |  |  |  |  |  |  |  |  |  |  |  |  |  |  |  | x(9) | x(10) |  |  |
| 3311 |  |  |  |  |  |  |  |  |  |  |  |  |  |  |  |  |  |  | **x(29)** |
| 3427 |  |  | x(8) |  |  |  |  |  |  |  |  |  |  |  |  |  |  |  |  |
| 3472 |  |  | x(7) |  |  |  |  |  |  |  |  |  |  |  |  |  |  |  |  |
| 3562 |  |  |  |  |  |  |  |  |  |  |  |  |  |  |  | x(4) |  |  |  |
| 3662 |  |  |  |  | x(4) |  |  |  |  |  |  | x(8) |  |  |  |  |  |  |  |
| 3695 |  |  |  |  |  |  |  |  | x(5) |  |  |  |  |  |  |  |  |  |  |
| 3708 |  |  |  |  |  |  |  |  | x(7) |  |  |  |  |  |  |  |  |  |  |
| 3766 |  |  |  |  |  |  |  |  |  |  |  | x(5) |  |  |  |  |  |  |  |
| 3801 |  |  |  |  |  |  |  |  | x(4) |  | x(5) |  |  | x(8) |  |  |  |  |  |
| 3822 |  | x(3) |  |  |  |  |  |  |  |  | x(5) |  |  |  |  |  |  |  |  |
| 3906 |  |  |  |  |  |  |  |  |  |  |  | **x(23)** |  |  | **x(15)** |  |  |  |  |
| 3929 |  |  |  |  |  |  |  |  |  |  | **x(25)** | **x(21)** | **x(23)** | **x(17)** | **x(25)** |  |  |  |  |
| 4063 |  |  |  |  |  |  |  |  |  |  |  |  |  |  |  |  |  | x(7) |  |
| 4184 | **x(16)** |  |  |  |  |  |  |  |  |  |  |  |  |  |  |  |  |  |  |
| 4194 |  |  |  |  |  |  |  |  |  |  |  |  |  |  |  |  |  |  | x(4) |
| 4212 |  | x(3) | x(7) |  |  |  |  |  |  | x(5) |  |  |  |  |  |  |  | x(7) |  |
| 4325 |  |  | x(7) |  |  |  |  |  |  |  |  |  |  |  |  |  |  |  |  |
| 4330 |  |  |  |  |  | x(9) |  |  |  |  |  |  |  |  |  |  |  |  |  |
| 4385 |  |  |  |  |  |  |  |  | x(9) |  |  |  |  |  |  |  |  |  |  |
| 4457 |  |  |  |  |  |  |  |  |  |  | x(9) | x(12) | x(12) | x(10) | x(13) | x(7) | x(6) |  |  |
| 4497 |  |  |  |  |  |  | x(10) | x(12) |  |  |  |  |  |  |  |  |  | **x(15)** |  |
| 4512 | x(5) |  |  |  | x(6) | x(7) |  |  |  | x(7) |  |  |  |  |  |  |  |  | x(13) |
| 4549 | x(4) |  |  |  | x(5) |  |  |  |  | x(10) |  |  |  |  |  |  |  |  |  |
| 4564 |  |  | x(14) |  |  |  |  |  |  |  |  |  |  |  |  |  |  |  |  |
| 4603 |  |  |  |  |  |  |  |  | x(4) |  |  |  |  |  |  |  |  | x(9) |  |
| 4642 |  |  |  |  |  |  |  |  |  |  |  |  |  |  |  | **x(22)** | **x(20)** |  |  |
| 4704 |  |  |  |  |  |  |  |  |  |  |  |  |  |  |  |  |  | x(9) |  |
| 4716 |  | x(3) |  |  |  |  |  |  |  |  |  |  |  |  |  |  |  |  | x(5) |
| 4939 |  |  |  |  |  |  |  |  |  |  |  |  |  | x(14) |  |  |  |  |  |
| 5022 |  | **x(17)** |  |  |  |  |  |  |  |  |  |  |  |  |  |  |  |  |  |
| 5041 |  | **x(32)** |  |  |  |  |  |  |  |  |  |  |  |  |  |  |  |  |  |
| 5064 |  | x(12) |  |  |  |  |  |  |  |  |  |  |  |  |  |  |  |  |  |
| 5091 |  |  |  |  |  |  |  |  |  |  |  |  |  |  |  |  |  |  | x(5) |
| 5109 | x(9) |  |  |  |  |  |  |  |  |  |  |  |  |  |  |  |  |  |  |
| 5205 |  |  |  | x(5) |  |  |  |  |  |  |  |  |  |  |  |  |  |  |  |
| 5263 |  |  |  |  |  |  |  |  | x(4) |  |  |  |  |  |  |  |  |  |  |
| 5323 |  |  |  |  |  |  |  |  |  |  |  |  | x(7) |  |  |  |  |  |  |
| 5340 |  |  |  |  |  |  |  |  |  |  |  |  |  |  | x(6) |  |  |  |  |
| 5425 | x(10) |  |  |  | **x(18)** | x(14) |  |  |  |  |  |  |  |  |  |  |  |  |  |
| 5452 |  |  |  | **x(26)** |  |  |  |  |  |  |  |  |  |  |  |  |  |  |  |
| 5510 |  |  |  |  | **x(27)** | **x(26)** |  |  |  |  |  |  |  |  |  |  |  |  |  |
| 5518 | x(21) |  |  |  |  |  |  |  |  |  |  |  |  |  |  |  |  |  |  |
| 5534 |  |  |  | x(14) |  |  |  |  |  |  |  |  |  |  |  |  |  |  |  |
| 5552 | x(13) |  |  |  |  |  |  |  |  |  |  |  |  |  |  |  |  |  |  |
| 5575 | x(9) |  |  | **x(15)** |  |  |  |  |  |  |  |  |  |  |  |  |  |  |  |
| 5674 |  |  |  |  |  |  |  |  |  |  |  |  |  |  |  | x(7) | x(10) |  |  |
| 5699 |  |  |  |  |  |  |  |  |  |  |  |  |  | x(5) | x(5) |  |  |  |  |
| 5821 |  |  |  |  |  |  |  |  |  | **x(21)** |  |  |  |  |  |  |  |  |  |
| 5917 |  |  |  |  |  |  |  |  |  |  |  |  |  |  |  | x(10) |  |  |  |
| 5973 |  |  |  |  |  |  |  |  |  |  |  |  |  |  |  |  |  | x(9) |  |
| 6026 |  |  |  |  |  |  |  |  |  | **x(25)** |  |  |  |  |  |  |  |  |  |
| 6039 |  | **x(18)** | **x(23)** |  |  |  |  |  |  |  |  |  |  |  |  |  |  |  |  |
| 6050 |  |  |  |  |  |  |  |  |  |  |  |  |  |  |  | x(10) |  |  |  |
| 6070 |  |  |  |  |  |  |  |  |  |  |  |  |  |  |  | x(8) |  |  |  |
| 6211 |  |  | x(8) |  |  |  |  |  |  |  |  |  |  |  |  |  |  |  |  |
| 6239 |  | x(4) |  |  |  |  |  |  |  |  |  |  |  |  |  |  |  |  | x(6) |
| 6312 |  |  |  |  |  |  |  |  |  |  |  |  |  |  | x(6) |  |  |  |  |
| 6396 |  |  |  |  |  |  |  |  |  | x(5) | x(6) |  |  |  |  |  |  |  |  |
| 6524 |  |  |  |  |  |  |  |  |  |  |  |  |  |  |  |  | **x(22)** |  |  |
| 6631 |  |  |  |  |  |  |  |  |  |  |  |  |  |  |  | **x(16)** | x(8) |  | **x(25)** |
| 7423 |  |  |  |  |  | x(10) |  |  | **x(24)** |  |  |  |  |  |  |  |  |  |  |
| 7435 |  |  |  |  |  |  |  |  |  |  |  |  |  |  |  | x(7) |  |  |  |
| 7461 |  |  |  |  |  |  |  |  | **x(27)** |  |  |  |  |  |  |  |  |  |  |
| 7556 |  |  |  |  |  |  |  | x(6) |  |  |  |  |  |  |  |  |  |  |  |
| 7735 |  |  |  |  |  |  |  |  | x(6) | x(6) |  |  |  |  |  |  |  |  | x(5) |
| 8012 |  |  |  |  |  |  |  |  |  | x(7) |  |  |  |  |  |  |  |  |  |
| 8156 |  |  |  |  |  |  |  |  |  |  |  |  |  |  |  |  |  | x(9) |  |
| 9213 |  |  |  |  |  |  |  |  |  |  |  |  |  |  |  |  |  | **x(20)** |  |
| 9659 |  |  |  |  |  | x(4) |  |  |  | x(4) |  |  |  |  |  |  |  | x(8) |  |
| 10112 |  |  |  |  |  | x(8) |  |  |  |  |  |  |  |  |  |  |  |  |  |
| 10488 |  |  |  |  |  |  |  |  |  |  |  |  |  |  | x(6) |  |  |  |  |
